# Adaptive Communication: Languages with More Non-Native Speakers Tend to Have Fewer Word Forms

**DOI:** 10.1371/journal.pone.0128254

**Published:** 2015-06-17

**Authors:** Christian Bentz, Annemarie Verkerk, Douwe Kiela, Felix Hill, Paula Buttery

**Affiliations:** 1 Department of Theoretical and Applied Linguistics, University of Cambridge, Cambridge, United Kingdom; 2 Reading Evolutionary Biology Group, School of Biological Sciences, University of Reading, Reading, United Kingdom; 3 Computer Laboratory, University of Cambridge, Cambridge, United Kingdom; Stony Brook University, UNITED STATES

## Abstract

Explaining the diversity of languages across the world is one of the central aims of typological, historical, and evolutionary linguistics. We consider the effect of *language contact*-the number of non-native speakers a language has-on the way languages change and evolve. By analysing hundreds of languages within and across language families, regions, and text types, we show that languages with greater levels of contact typically employ fewer word forms to encode the same information content (a property we refer to as *lexical diversity*). Based on three types of statistical analyses, we demonstrate that this variance can in part be explained by the impact of non-native speakers on information encoding strategies. Finally, we argue that languages are information encoding systems shaped by the varying needs of their speakers. Language evolution and change should be modeled as the co-evolution of multiple intertwined adaptive systems: On one hand, the structure of human societies and human learning capabilities, and on the other, the structure of language.

## Introduction

All languages are carriers of information. However, they differ greatly in terms of the encoding strategies they adopt. For example, while in German a single compound can transmit complex concepts (e.g. *Schifffahrtskapitänkabinenschlüssel*), English uses whole phrases to transmit the same information (*key to the cabin of the captain of a ship*). In the Eskimo-Aleut language Inuktitut the word *qimmiq* ‘dog’ can be modified to encode different case relations, e,g. *qimmi-mik* ‘with the dog’, *qimmi-mut* ‘onto the dog’, *qimmi-mi* ‘in the dog’, *qimmi-mit* ‘away from the dog’, etc [1]. Likewise, many languages encode information about number, gender and case in a multitude of different articles, e.g. German *der, die, das, dem, den, des* or Italian *il, la, lo, i, le, li, gli*, whereas in English there is only one definite article *the* and in Mandarin Chinese there is none.

We refer to this property of languages—the distribution of word forms or *word types* they use to encode essentially the same information—as their *lexical diversity* (LDT). This difference is a central part of the variation in encoding strategies we find across languages of the world.

This paper centers on the question of where variation in lexical diversity stems from. Why do some languages employ a wide range of opaque lexical items while others are more economical? Variation between languages has often be seen as driven by language acquisition of native speakers (L1) [2–8]. However, some sociolinguistic and historical studies have raised the question of whether large numbers of non-native (L2) language speakers in a society can also lead to systematic changes in the use of the language in generall [9–16].

In this work we investigate with quantitative analyses the association between non-native language speaker proportions—here referred to as *language contact*—and variation in lexical diversity. Adults learning a second language encounter difficulties with the panoply of word forms that native speakers seem to master with ease, so that non-native language is typically characterised by lower lexical diversity [17, 18]. We consider whether higher proportions of non-native speakers in a population should over time reduce the lexical diversity of a language. A clear prediction of this hypothesis is that, at any point in time, languages with higher L2 speaker proportions are those languages that have lower lexical diversities.

To systematically compare lexical diversities cross-linguistically we use parallel translations of the same texts into hundreds of languages. Parallel translations provide a natural means of controlling for constant information content. The LDT of these texts can be quantified by applying three measures: the parameters of the Zipf-Mandelbrot law [19, 20], Shannon entropy [21, 22] and type-token ratios [23–26]. Using these measures, we observe a great variety of lexical diversities across language families and regions of the world despite constant content of the texts.

To test whether some of this variation can be attributed to language contact, we employ three types of statistical model: a) simple linear regression, regressing lexical diversities on L2 speaker proportions; b) linear mixed-effects regression controlling for family relationships, regional clustering and text type; and c) phylogenetic generalized least squares regression (PGLS) that models the potential co-evolution of L2 speaker proportions with lexical diversities. The results of these models converge to show that the ratio of non-native speakers predicts lexical diversity beyond language families, regional clustering and text types.

These results can be interpreted as an example of a co-evolution between sociolinguistic niches (more or less non-native influence) and language structure (lower or higher lexical diversity) [12, 27]. From this perspective languages are complex adaptive systems shaped by the communicative needs and learning constraints of speaker populations [28–33]. We conclude that lexical diversity is a quantitative linguistic measure which is highly relevant to the enquiry of language evolution, language typology and language change, and that it can be modeled taking into account sociolinguistic and genealogical information. This supports the claim that the evolution of language structure can only be understood as a co-evolution of population structure, human cognitive constraints and communicative encoding strategies.

## Materials and Methods

### Parallel texts

The parallel texts used in this study are the *Universal Declaration of Human Rights* (UDHR) in unicode (http://www.unicode.org/udhr/), the *Parallel Bible Corpus* (PBC) [34] and the *Europarl Parallel Corpus* (EPC) [35].

The UDHR currently comprises a collection of more than 400 parallel translations. However, only 376 of these are fully converted into unicode. The UDHR is a short legal text of 30 articles and ca. 1700 words in English.

The PBC is a collection of parallel translations of the Bible. It currently comprises 918 texts that have been assigned 810 unique ISO 639-3 codes (i.e. unique languages). Texts are aligned by verses, which allows us to fully parallelize them by including only the verses that occur in all the texts of the respective language sample we are looking at. Note, that there is a trade-off between number of texts and number of verses. Not a single verse is represented in all texts. We chose a sample of 800 texts which yields overlapping verses that amount to ca. 20000 words in the English translation. This sample represents 632 languages (unique ISO 639-3 codes).

The EPC is a collection of transcripts of discussions in the European Parliament in 21 European languages. The English transcripts amount to ca. 7 million words.

Combining the UDHR, PBC and EPC yields a sample of 867 texts with 647 unique ISO 639-3 codes representing languages (see S1 Table). These languages stem from 83 families and 182 genera according to the *World Atlas of Language Structures* (WALS) classification [36].

### Defining word types

Any measure of lexical diversity relates to the frequency of occurrence of word types in a given text. A *word type* is here defined as a recurring sequence of letters delimited by white spaces, punctuation marks, and other non-word characters.

Note, that this definition of a word type rules out pictographic and logographic writing systems (see S1 File). Also, this simplified definition of “word” is contested by linguistically more informed approaches [37, 38]. However, to our knowledge it is currently the only computationally feasible approach for automatically generating lists of word types across hundreds of languages.

### Lexical diversity measures

**Fig 1:**
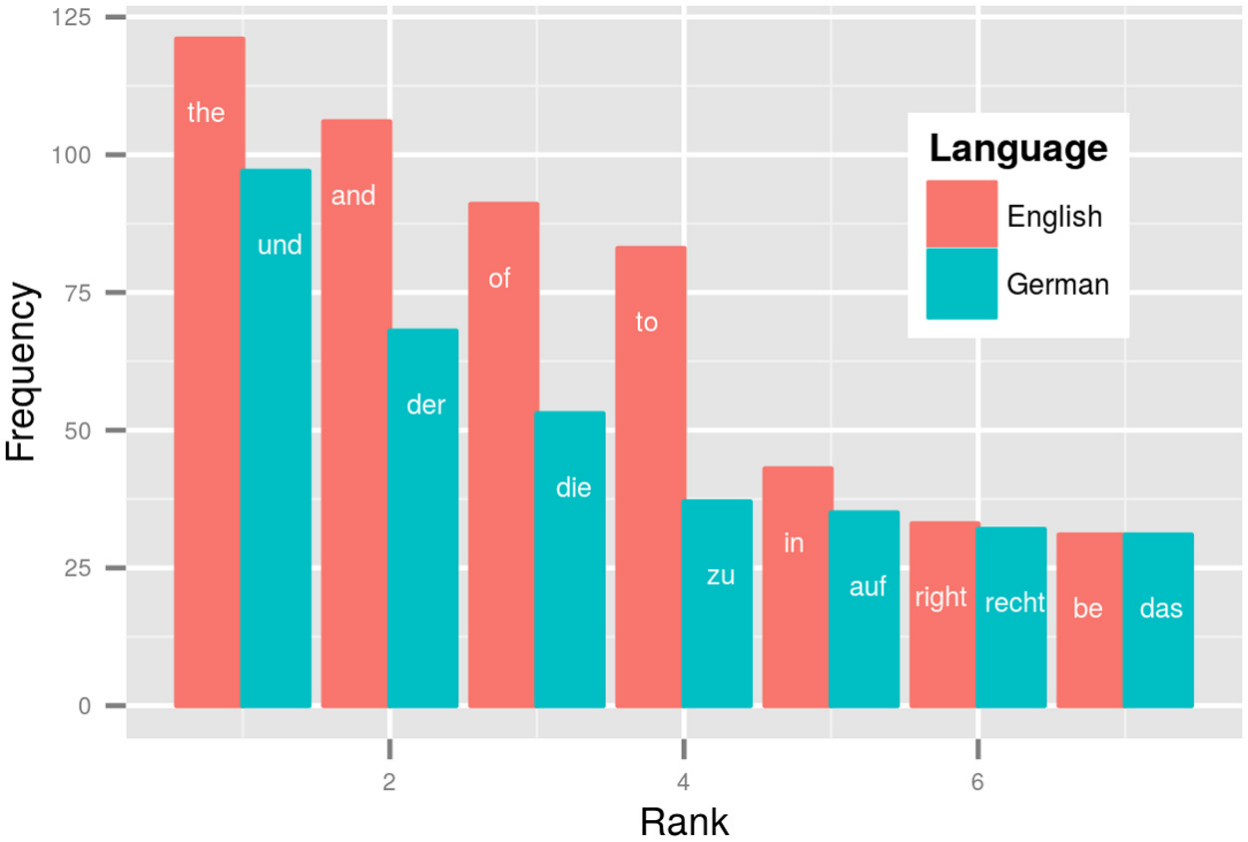
Word frequency distributions for English and German UDHR. Bars indicate frequencies of occurrence in German (blue) and English (red) for the highest ranking words in the UDHR text.

To scrutinize the distribution of word types in a given text they are ordered according to their frequency of occurrence. For example, Fig 1 displays the first 7 ranks of word types with their frequencies for the UDHR in German and English. Despite the constant information content of these parallel translations, English’s repetitive usage of the same word types results in high frequencies in the upper ranks. For example, the letter sequence representing the definite article in English (*the*) occurs roughly 120 times in the English UDHR, whereas German distributes occurrences of articles over different word types, i.e. *der* (ca. 60), *die* (ca. 50) and *das* (ca. 30).

To facilitate an investigation of varying lexical diversities across languages we introduce three quantitative measures of LDT: The parameters of Zipf-Mandelbrot’s law, Shannon entropy, and type-token ratios.

#### Zipf-Mandelbrot’s law

**Fig 2:**
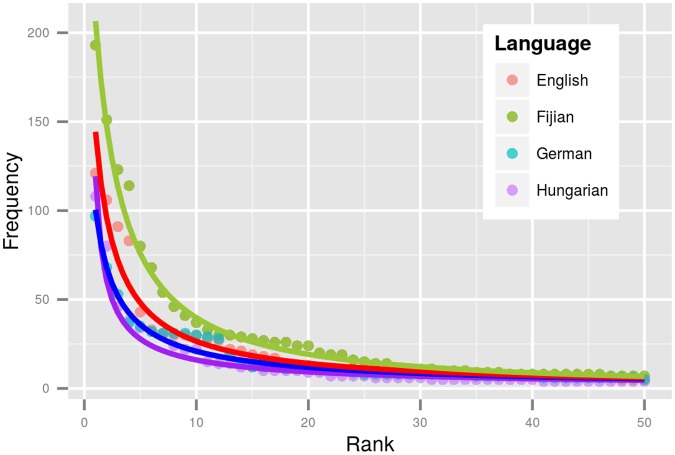
Word frequency distributions with ZM parameter approximations for selected languages. Dots represent frequencies and ranks for the 50 highest frequency words in English (red), Fijian (green), German (blue) and Hungarian (purple). Lines reflect Zipf-Mandelbrot approximations. Lower frequencies towards the first ranks are associated with more word types in the tails of distributions. More diverse languages have more hapax legomena (i.e. words with frequency = 1), i.e. Hungarian has more hapax legomena than German, English, and Fijian, in this order.

The shape of word frequency distributions can be approximated by the Zipf-Mandelbrot curve [19].

$$\begin{array}{c} f\left(r\right)=\frac{C}{{\left(\beta +r\right)}^{\alpha }}\;C>0,\;\alpha >0,\;\beta >-1,\;r\in {\mathbb{R}}^{+}, \end{array}$$ (1)

where *f(r)* is the frequency of a word in rank *r*, *α* and *β* are parameters and *C* is a normalizing constant. The parameters specify the shape of the approximated distribution. They can be estimated for individual languages by using a maximum likelihood estimation procedure (see S2 File). The lines in Fig 2 represent such approximations for Fijian, English, German and Hungarian. Notably, the Fijian approximation has the highest values (*C* = 0.39, *β* = 2.07, *α* = 1.2) and Hungarian the lowest values (*C* = 0.06, *β* = -0.33, *α* = 0.76), with German and English in between (see Fig 2).

There is an inverse relationship between lexical diversity and the ZM-parameters: lower diversity is associated with higher values for *C*, *α* and *β*.

#### Shannon entropy

Another measure for LDT is the entropy *H*_*w*_ over a distribution of words calculated as [21][p. 19]:

$$\begin{array}{c} H_{w}=-K\sum_{i=1}^{k}p_{i}\times log_{2}\left(p_{i}\right), \end{array}$$ (2)

where *K* is a positive constant determining the unit of measurement (which is bits for *K* = 1 and log to the base 2), *k* is the number of ranks (or different word types) in a word frequency distribution, and *p*_*i*_ is the probability of occurrence of a word of *i* ^*th*^ rank (*w*_*i*_).

The Shannon entropy in Eq 2 is a measure of the overall uncertainty when we draw words randomly from a text. A lexically diverse language such as Hungarian has more word types with lower frequencies. To put it differently, if we select a word at random from a Hungarian text and have to guess which word this is, the overall uncertainty is higher compared to a language with fewer word types and higher frequencies, such as English. Shannon entropy can therefore be used as an index for LDT, in parallel to the entropy index for biodiversity [39]. In particular, higher entropies of word frequency distributions are associated with higher LDT.

#### Type-token ratios

Finally, the most basic measure of lexical diversity is the so-called type-token ratio (TTR). TTR simply represents the number of different word types divided by the overall number of word tokens. Higher TTRs reflect higher lexical diversity. Note, that TTRs have been criticized as a measure of lexical diversity, since they are strongly dependent on text size [23, 25, 40]. However, in the case of parallel texts, information content is constant. Therefore, in the present analyses variation in text size is not a confound, but rather a crucial part of the differences in lexical encoding strategies that we aim to measure.

#### Differences between the measures

While Zipf-Mandelbrot parameters, Shannon entropy and type-token ratios are all measures that reflect LDT, there are important differences. ZM-parameters are negatively correlated with LDT (higher parameter values mean lower lexical diversity), whereas both entropy and TTRs exhibit a positive relationship with LDT. Less evidently, the “responsiveness” of these measures to changes in word frequency distributions varies somewhat. As we show in the supporting information (S3 File), TTR is the most responsive and hence fast changing measure, whereas Zipf-Mandelbrot’s *α* and Shannon entropy *H*_*w*_ are more conservative, in this order. However, to our knowledge there is no a priori reason to prefer one measure over the others. Hence, we calculate values for each of them and include them in our analyses.

#### Scaling of LDT measures

Since ZM’s *α* is negatively correlated with LDT, whereas *H*_*w*_ and TTR are positively correlated with LDT, we inverse ZM’s *α* by substracting the values from 1. Additionally, we scale the LDT values using the *scale()* function in *R* [41]. By default, this centeres and scales a vector of LDT values dividing it by the standard deviations per measure *m* and text *t*:

$$\begin{array}{c} LDT_{scaled}=\frac{LDT}{\sigma_{mt}}=\frac{LDT}{\sqrt{\frac{\sum {\left(LDT-\mu_{mt}\right)}^{2}}{\left(n_{mt}-1\right)}}}. \end{array}$$ (3)

This way, we combine the values for *α*, *H*_*w*_ and TTR into a single, scaled LDT measure. Note that different parallel corpora vary in text sizes, which in turn influences LDT values. Scaling these values makes them commensurable across text sizes. The scaled LDT is then used as dependent variable for statistical modeling.

### Non-native speaker data

Our dataset of speaker information contains languages for which we could obtain the numbers of native (L1) and non-native (L2) speakers in the linguistic community. We were able to collect this speaker information for 226 languages using the *SIL Ethnologue* [42], the *Rosetta project website* (www.rosettaproject.org), the *UCLA Language Materials Project* (www.lmp.ucla.edu), and the *Encarta* (http://en.wikipedia.org/wiki/Encarta).

We define L2 speakers as adult non-native speakers as opposed to early bilinguals. Generally, the sources follow our L2 definition, although in some cases the exact “degree” of bilingualism might vary (see, e.g., “bilingualism remarks” in Ethnologue).

Whenever native and non-native speaker numbers differed in the sources, we calculated the average. Note, that this averages out some of the incommensurable values that are certainly to be found in sources like Ethnologue. For example, English has 505 million L2 users world wide according to Ethnologue, whereas for German only L2 users within Germany are counted, which amounts to 8 million. Though English arguably has more L2 speakers than German, the difference is probably too big here. However, averaging across different sources in our data sample we arrive at 365 million L2 speakers for English and 50 million L2 users for German, which seems much more realistic (see S2 Table).

Note that we excluded Sanskrit and Esperanto from the sample. Sanskrit is an extreme outlier in the Indo-European family. In our database it is listed with a very high ratio of L2 to L1 speakers. This is due to the fact that it is learned and used almost exclusively as liturgical language in Hinduism. In this sense, there are very few native speakers of Sanskrit but many that learn it in schools as L2 for liturgical purposes. Clearly, this is not the kind of L2 learning and usage scenario that is supposed to reduce lexical diversity. Esperanto, on the other hand, is an artificial language with a high ratio of L2 speakers. However, since it is a constructed language there is no point to be made about potential shaping of its linguistic structure due to natural processes of language change (though there might be such processes at play in its very recent history).

Based on the remaining averaged speaker numbers we then calculated the ratio of L2/L1 speakers for each of the 226 languages. This serves as our main predictor variable in the statistical models.

### Statistical models

#### Linear regression

To explore a potential association between lexical diversity and L2 speaker proportions we first merge the data on scaled LDTs (647 languages) with the data on L2 ratios (226) languages. This yields a sample of 91 languages (26 different families) (see S2 Table for the full data set). We then construct a simple linear model with the scaled LDT measure as response variable and the ratio of non-native (L2) to native (L1) speakers as predictor variable. L2 speaker ratios are logarithmically transformed to reduce extreme outliers. The model is outlined in Eq 4:

$$\begin{array}{c} LDT=\beta_{0}+\beta_{1}\times log\left(L2\right)+\epsilon ,\\ \epsilon ∼N(0,\sigma^{2}). \end{array}$$ (4)

The lexical diversity *LDT* is predicted by the intercept *β*_0_ plus the slope *β*_1_ multiplied by the logarithm of the ratio of L2 to L1 speakers (here represented by *L*2), and the error *ϵ*. One of the underlying assumptions of a linear regression model is that the errors are normally distributed between 0 and the variance *σ* ^2^. Likewise, the assumption of *linearity* and *homoscedasticity* have to be met for the model to be valid. Post hoc checking of these assumptions can be found in the supporting information (S4 File).

We use the function *lm()* in *R* [41] for building this linear model.

#### Linear mixed-effects regression

Language typologists have suggested that simple linear models are undermined by the non-independence of data points. Namely, languages naturally group into families and regions [43, 44]. Moreover, we draw texts from three different sources, use three different measures of LDT and hence have multiple LDT values per ISO 639-3 code. These groupings can introduce systematic variation. Such grouped data require modeling by means of mixed-effects models [45, 46].

Hence, we expand the simple linear model by introducing (non-correlated) intercepts and slopes by family, region, LDT measure, text type and ISO 639-3 code. Information on language families and language regions is taken from Bickel and Nichol’s AUTOTYP database (www.spw.uzh.ch/autotyp/).

The mixed-effects model specification can be found in Eq 5:

$$\begin{array}{c} LDT_{frmti}=\beta_{0}+F_{0f}+R_{0r}+M_{0m}+T_{t0}+I_{i0}+\\ \left(\beta_{1}+F_{1f}+R_{1r}+M_{1m}+T_{1t}+I_{1i}\right)\times log\left(L2_{frmti}\right)+\epsilon_{frmti},\\ \epsilon_{frmti}∼N(0,\sigma^{2}). \end{array}$$ (5)

Here, *LDT*_*frmti*_ is the predicted lexical diversity for languages of the *f* ^*th*^ family, *r* ^*th*^ region, *m* ^*th*^ measure and *t* ^*th*^ text type and *i* ^*th*^ ISO 639-3 code. The coefficients *β*_0_ and *β*_1_ represent the fixed effects intercept and slope respectively. *F*_0*f*_, *R*_0*r*_, *M*_0*m*_, *T*_0*t*_, *I*_*i*0_ are the random intercepts by family, region, measure, text type and ISO code. *F*_1*f*_, *R*_1*r*_, *M*_1*m*_, *T*_1*t*_, *I*_1*i*_ denote random slopes by family, region, measure, text type and ISO code. The linear predictor is the log-transformed L2 ratio (*L*2_*frmti*_). Model residuals are represented by *ϵ*_*frmti*_. Again, residuals are supposed to be normally distributed between 0 and their variance *σ* ^2^.

Again, the models are run in *R* [41] using the package *lme4* [47]. As for the simple linear model, we check for *linearity*, *normality* and *homoscedasticity* in the supporting information (S4 File).

#### Phylogenetic analyses

The Mixed-effects model tests whether the statistical association between L2 ratio and lexical diversity holds even if systematic differences *between* language families are accounted for. However, we could also ask if the patterns we find hold *within* language families, namely at the level of genera and sub-genera (e.g. Romance and Germanic languages within the Indo-European family). The dataset of L2 speakers and lexical diversities is currently too small to run a mixed-effects model with genera as random effects, since there are very few genera with more than 5 representatives. Instead, phylogenetic regressions [48–50] can be used to assess whether lexical diversities of extant languages are driven by differences in the ratios of L2 speakers while taking into account their genealogical relationships.

We first use published linguistic family trees [51–53] based on cognate lists as a measure of genealogical relationships. The tips of these phylogenetic trees represent extant languages. The nodes within the trees reflect ancestral languages, and their branches reflect the evolutionary pathways that individual languages have taken.

We can assess the likelihood of whether the lexical diversities of extant languages followed closely the evolutionary pathways given in the tree (high “phylogenetic signal”) or whether this tree has to be strongly reduced to fit the lexical diversity data (low “phylogenetic signal”) [48]. On the basis of the phylogenetic signal analysis, we can then use *phylogenetic generalized least squares* (PGLS) regression to test whether L2 ratio is still a significant predictor of LDT if we correct for the co-variance within the family.

#### Phylogenetic signal

To establish whether lexical diversities evolve along the phylogenetic branches of family trees, a test for phylogenetic signal called *λ* (lambda) is employed. The estimation of *λ* is a phylogenetic comparative method that transforms a phylogenetic tree to best fit the comparative data [48, 49]. Namely, *λ* is a factor that modifies the branch lengths of phylogenetic trees so that they fit the comparative data of interest. The *λ*-values can range from 0 to 1, with 1 meaning that the similarities in LDT can be explained by their relationship on the phylogeny; while 0 means that there is no evidence for similar behaviour due to shared decent.

Note, that for the phylogenetic analyses we need to link a single ISO code to both the phylogenetic tree information, and to the respective LDT information. A dataset with doubled ISO codes is not workable. Hence, analyses have to be run by LDT measures and text types separately. Moreover, since we do *within* family analyses, there need to be LDT data available for at least 20 languages in the family tree [48]. Given these restrictions, the phylogenetic signal *λ* is estimated for data from three different language families: Austronesian, Bantu and Indo-European (see Table 1 for the datasets used).

**Table 1:** Data sets for phylogenetic signal analyses.

**Family**	**Text**	**No. languages**	**Phylogenetic tree set**	**Size of tree set**
Austronesian	UDHR	28	Gray et al.(2009)	1000
Austronesian	PBC	44	Gray et al.(2009)	1000
Bantu	UDHR	26	Grollemund et al. (to appear)	100
Indo-European	UDHR	53	Bouckaert et al. (2012)	1000 (random sample from original 12500)

#### Phylogenetic generalized least squares regression

To illustrate the association between lexical diversity and the ratio of non-native (L2) speakers within families while controlling for phylogenetic signal, *Phylogenetic Generalized Least Squares* (PGLS) regressions [50] are carried out for Indo-European languages of the UDHR. This is currently the only family represented by enough languages with information on L2 speakers to run such a PGLS.

The phylogenetic tree used for the PGLS regression is a 1000 tree subsample of an earlier study [53]. Matching the dataset on LDT values and ratio of non-native speakers with the languages featured in the tree sample yields a sample of 26 Indo-European languages for PGLS regression analysis (see Table 2).

**Table 2:** Data set for PGLS regression.

**Family**	**Text**	**No. languages**	**Phylogenetic tree set**	**Size of tree set**
Indo-European	UDHR	26	Bouckaert et al. (2012)	1000 (random sample from original 12500)

As dependent variables the LDT measures ZM’s *α*, Shannon entropy *H*_*w*_, and TTR are used separately. The predictor variable is ratio of L2 speakers as before. PGLS regression was conducted using *Continuous* implemented in the software *BayesTraits* [49, 54], which uses a Bayesian reversible-jump Markov chain Monte Carlo framework to model and test hypotheses regarding the evolution of biological and linguistic traits (see S5 File). The MCMC chains were run for 2 × 10^9^ iterations for all three analyses. The PGLS estimates were sampled every 10^6^ iterations. A posterior of 1500 samples was taken from the stationary part of the chain.

#### Multiple testing: The Holm-Bonferroni correction

For the phylogenetic generalized least squares regressions we use three LDT measures and hence conduct multiple tests. To correct for multiple testing we use the Holm-Bonferroni correction [55]. According to that method, p-values are first ordered from lowest to highest. Then the *α*-level of significance (i.e. 0.05) is divided by the number *m* of tests (3 in our case). The lowest p-value has to be below this modified level (i.e. 0.05/3 = 0.017), the next lowest p-value has to be below the level of (0.05/m-1 = 0.025), the last p-value has to be below the original *α*-level of 0.05. All p-values that are significant according to the Holm-Bonferroni method will be marked by a star.

## Results

### Lexical diversities across 647 languages

**Fig 3:**
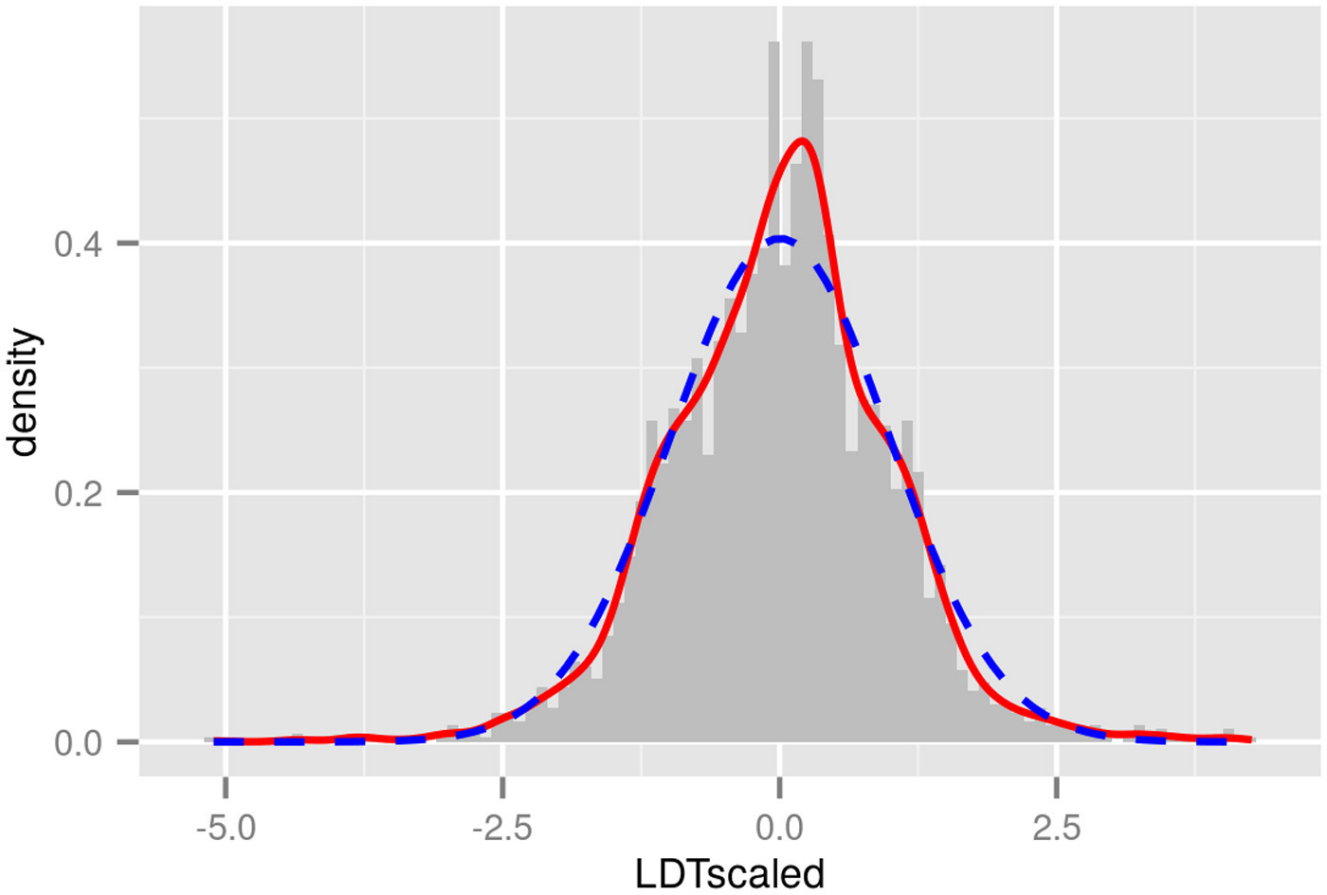
Lexical diversity distribution. Scaled LDT measures for 647 languages (histogram with grey bars), with smoothing function overlaid (red). The corresponding normal distribution is plotted in blue (dashed line).

Recall that our text sample comprises 846 parallel translations representing 647 unique languages of 83 different language families. The scaled LDT measures for all of these languages range from -5.11 to 4.26 and roughly follow a normal distribution (Fig 3).

Among the outliers with highest LDT values are Cherokee (chr), Finnish (fin), Inuktitut (ike), varieties of Quechua (quh, quy, quc), and Zulu (zul). Among the languages with lowest LDT values are Hmong (hea), Pidgin Nigerian (pcm) and Vietnamese (vie).

**Fig 4:**
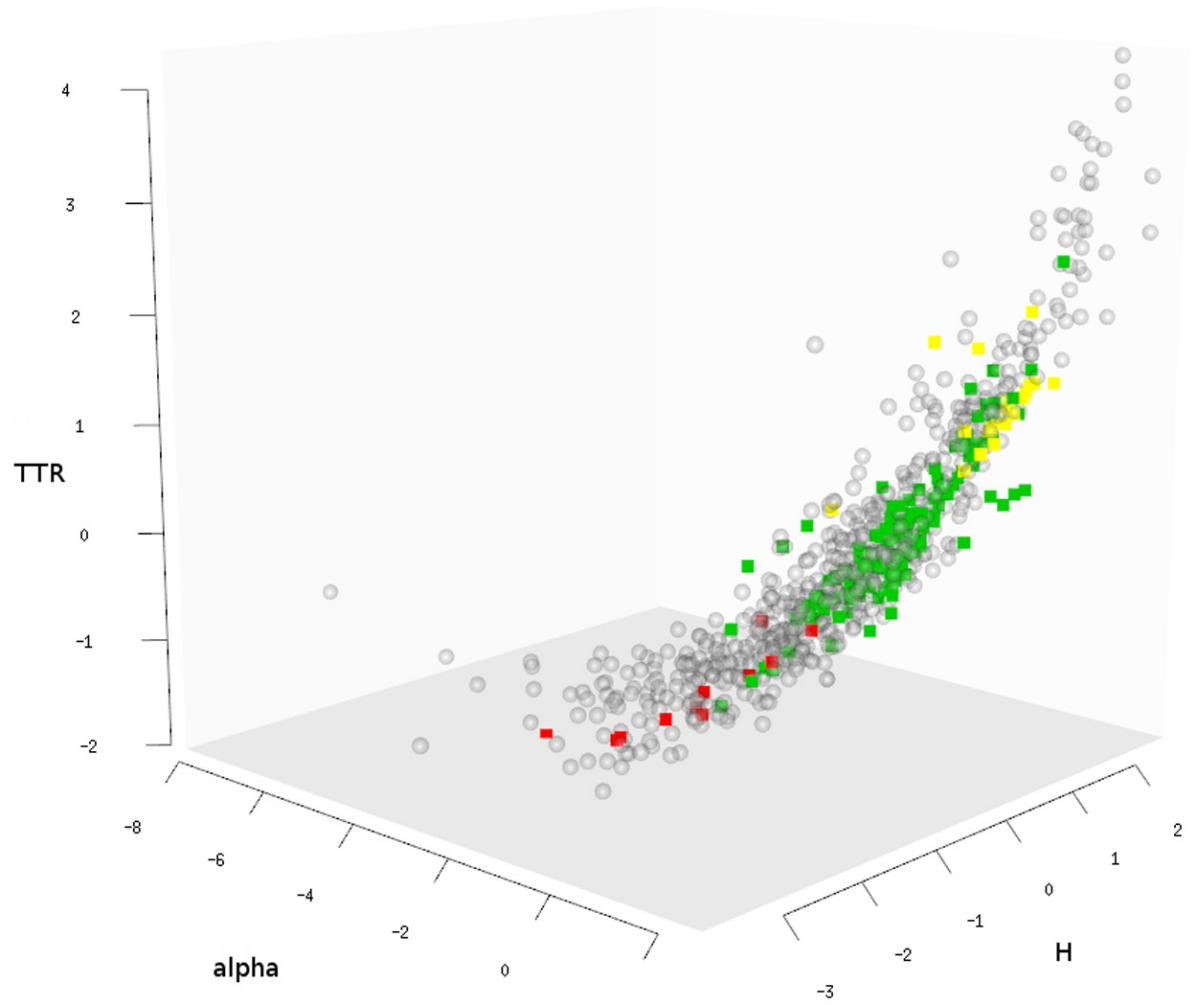
Lexical diversity space. Locations of 647 languages along ZM’s *α*, *H*_*w*_ and TTR (centered and scaled). Highly diverse languages cluster towards the upper-right corner in the back (highest values), whereas lexically redundant languages cluster towards the lower-left corner in the front (lowest values). To illustrate between-family variation, Altaic (yellow squares), Indo-European (green squares) and Creole languages (red squares) are pointed out among languages of other families (grey dots).

To visually illustrate the range of values for all languages and all three LDT measures, we plot each language as a point in a three dimensional “lexical diversity space” along the dimensions of ZM’s *α*, *H*_*w*_ and TTR (see Fig 4).

It is apparent that there is systematic LDT variation *between families*. For example, Altaic languages (Turkish, Azerbaijani, Kazakh, Uzbek, etc.) have high *α*, *H*_*w*_ and TTR values, cluster together in the upper-right corner (yellow squares), and hence display high lexical diversity. On the contrary, Creole languages have low *α*, *H*_*w*_ and TTR values, cluster in the lower-left corner, and display low lexical diversity (red squares). Indo-European languages range somewhere in between (green squares).

**Fig 5:**
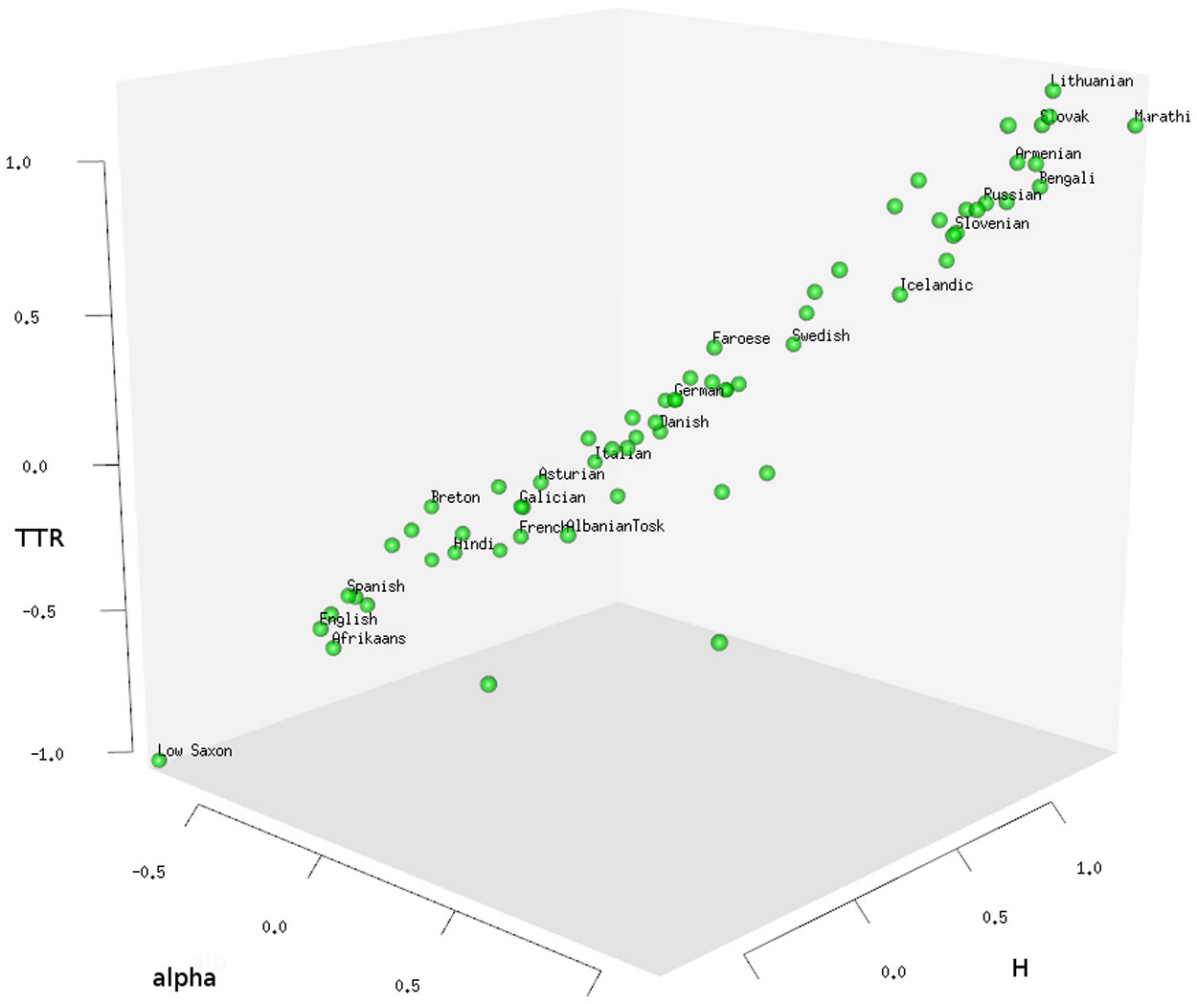
Lexical diversity space for Indo-European languages. Locations of Indo-European languages along ZM’s *α*, *H*_*w*_ and TTR (UDHR only). High LDT languages are to be found in the upper-right corner (e.g. Lithuanian, Marathi), low LDT languages are to be found in the lower-right corner (e.g. Low Saxon, English, Afrikaans).

On top of *between-family* variation, there is also *within-family* variation in our data sample. This is illustrated for Indo-European languages of the UDHR in Fig 5. Even within the same familiy (Indo-European) there is a considerable spectrum of LDT values, ranging from Low Saxon (nds), on the extreme low end, to Marathi (mar), at the high end.

Our working hypothesis is that this *between* and *within* family variation can partly be explained by individual histories of language contact, i.e. the ratio of non-native to native speakers per language.

### Linear regression

**Fig 6:**
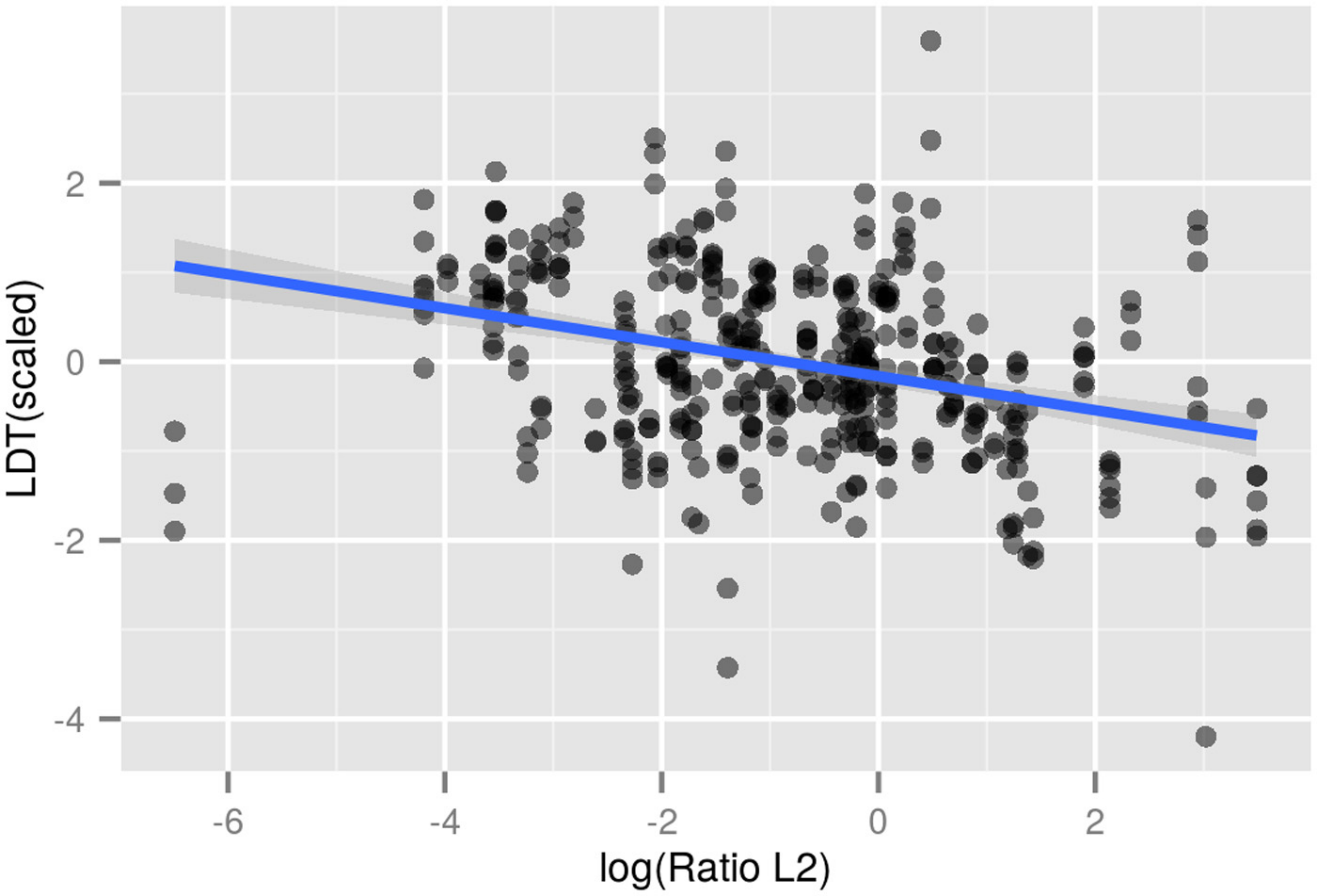
Linear regression. Linear model for the relationship between the ratio of L2 speakers versus L1 speakers (logarithmically transformed) and scaled lexical diversities. Model parameters (*β*-coefficients, *R* ^2^-values and t-values are displayed in Table 3). The blue line indicates a linear model with the respective intercept and slope (coefficient) and 95% confidence intervals.

For our sample of 91 languages, a linear regression with the logarithm of L2 ratios as predictor and the scaled LDT measure as dependent variable suggests that languages with higher L2 ratios have lower LDTs (Fig 6 and Table 3).

**Table 3:** Results for linear regression model.

**Dep. var.**	**Indep. var.**	**R^2^**	**coefficient**	**SE**	**t-value**	**p-value**
LDT (scaled)	log(L2/L1)	0.1109	-0.19051	0.02592	-7.349	1.04e-12

Namely, there is a negative coefficient (i.e. slope) of -0.19 between the linear predictor of L2/L1 ratio and the LDT of languages. This means that an increase of L2/L1 ratio by one unit is corresponding to a decrease of LDT by 0.19. This is a moderate, but strongly significant effect considering that the absolute range of scaled LDT values is ca. 8. Accordingly, the variance in LDT explained by the model (*R* ^2^) amounts to ca. 11%.

### Linear mixed-effects regression

For the same 91 languages the linear mixed-effects regression controlling for families, regions, measures, text types and ISO codes yields a similar result, as can be seen in Table 4. Again, the coefficient is negative (ca. -0.28) and significantly different from zero.

**Table 4:** Results for linear mixed-effects regression.

**Dep. var.**	**Fixed eff.**	**Random eff.**	**coefficient**	**SE**	**t-value**	**p-value**
LDT (scaled)	log(L2/L1)	family, region, measure, text, ISO code	-0.2772	0.1329	-2.087	0.0375

**Fig 7:**
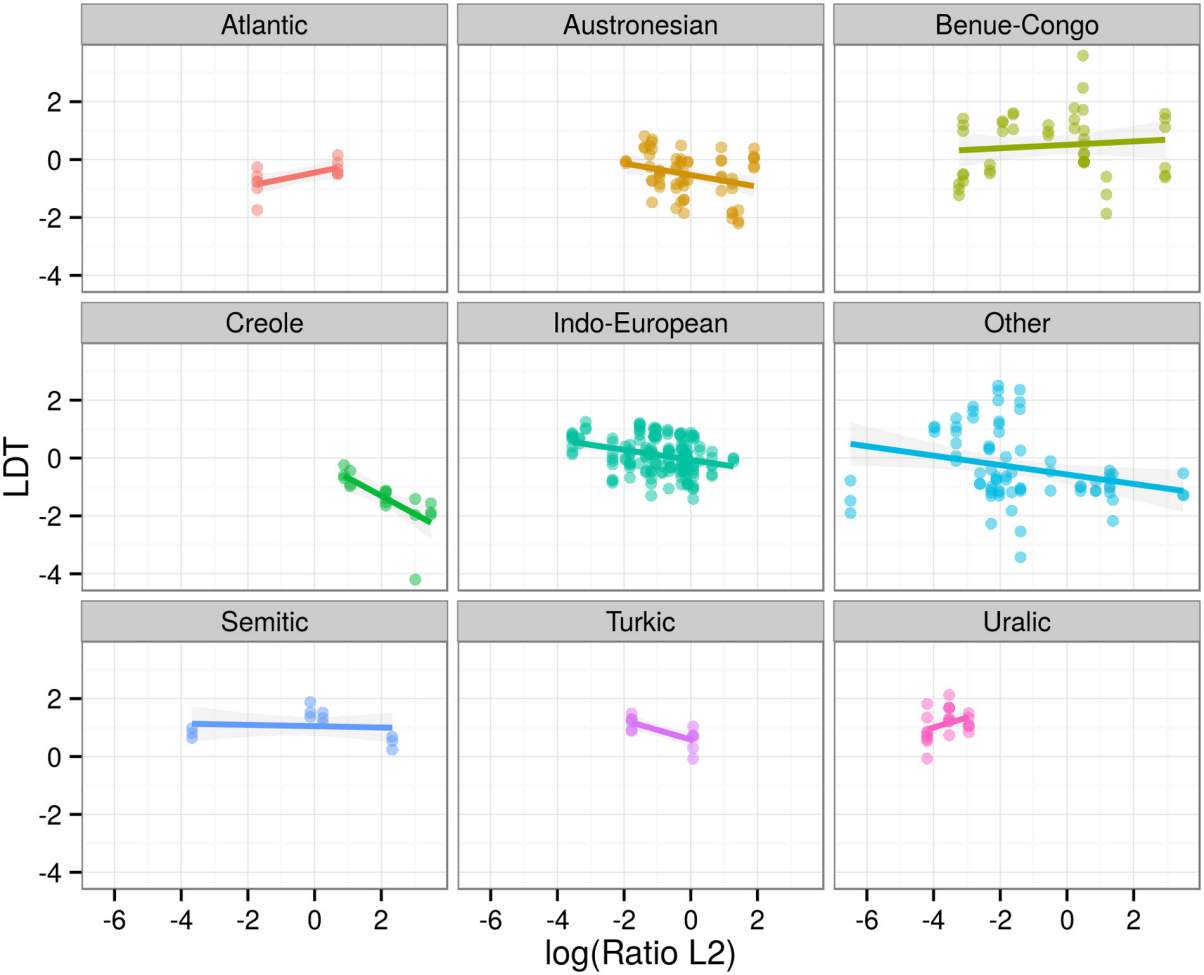
Regression plots by families. Scatterplots of log-transformed ratios of L2 speakers versus LDTs facetted by language families. Colored lines represent linear models by families with 95% confidence intervals. Languages of families with less than 10 data points are subsumed under “Other”. Note that this is just done for plotting, for statistical modeling language families are not collapsed.

**Fig 8:**
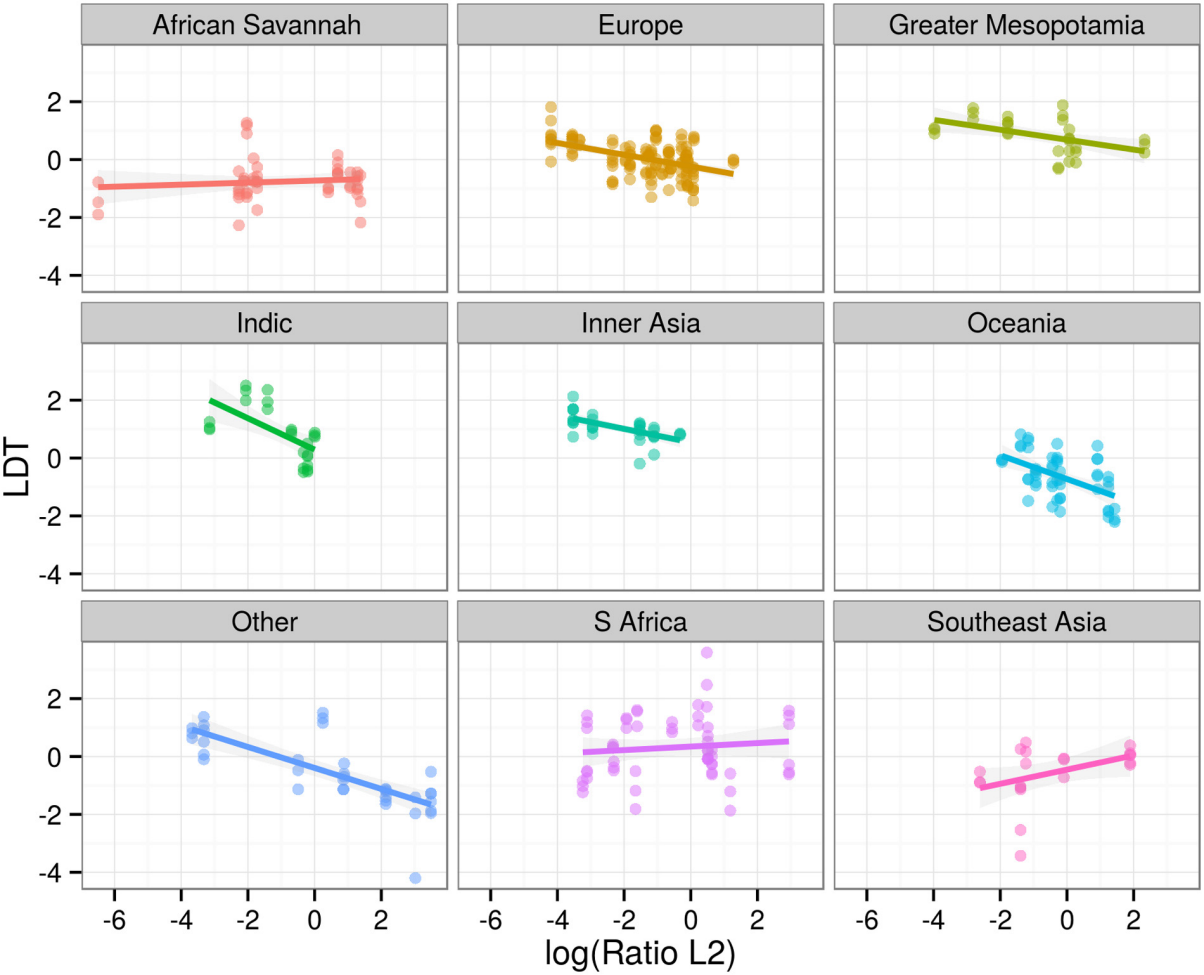
Regression plots by regions. Scatterplots of log-transformed ratios of L2 speakers versus LDTs facetted by language regions. Colored lines represent linear models by families with 95% confidence intervals. Languages of regions with less than 10 texts are subsumed under “Other”. Note that this is just done for plotting, for statistical modeling language regions are not collapsed.

**Fig 9:**
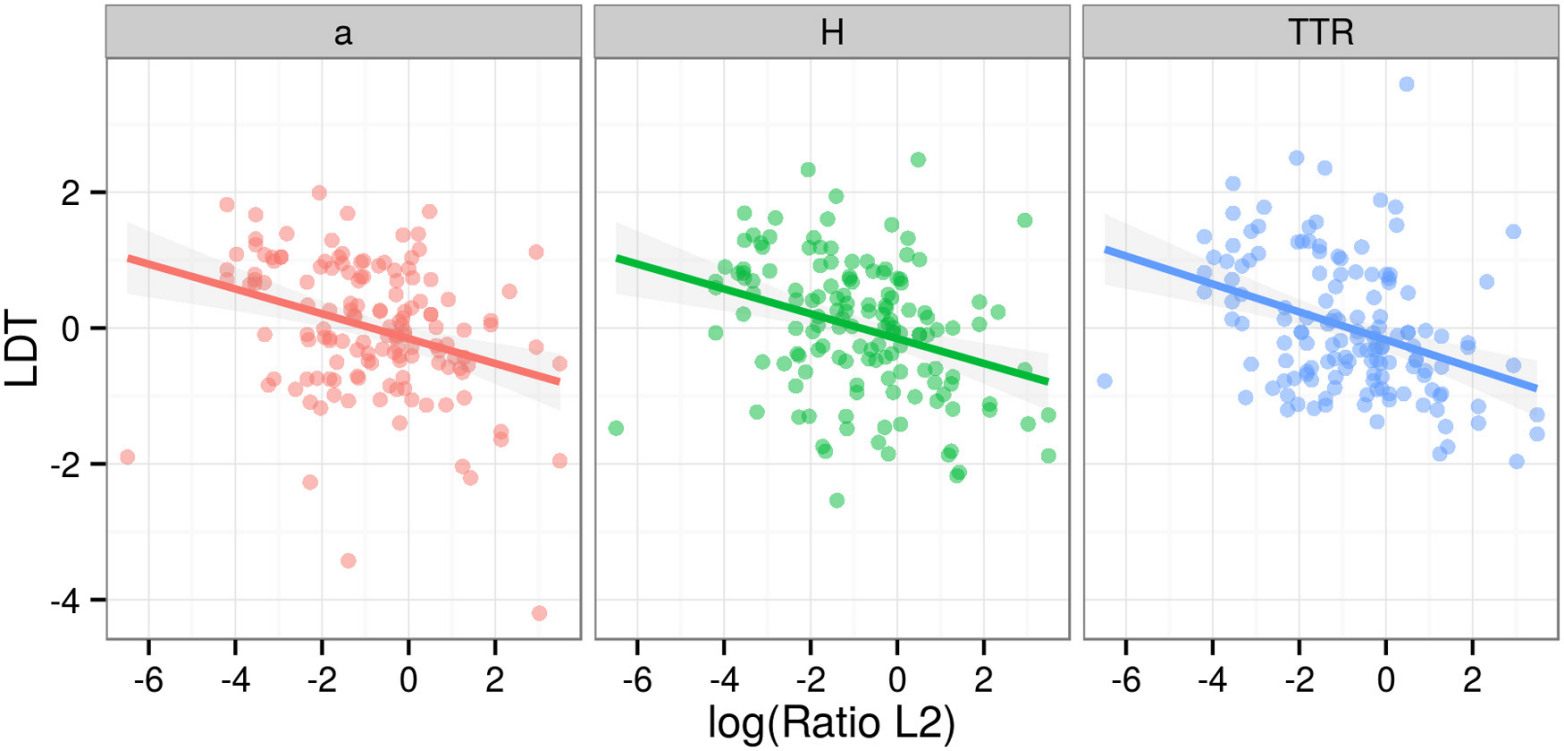
Regression plots by LDT measures. Scatterplots of log-transformed ratios of L2 speakers versus LDTs facetted by LDT measures. Lines represent linear models by families with 95% confidence intervals.

**Fig 10:**
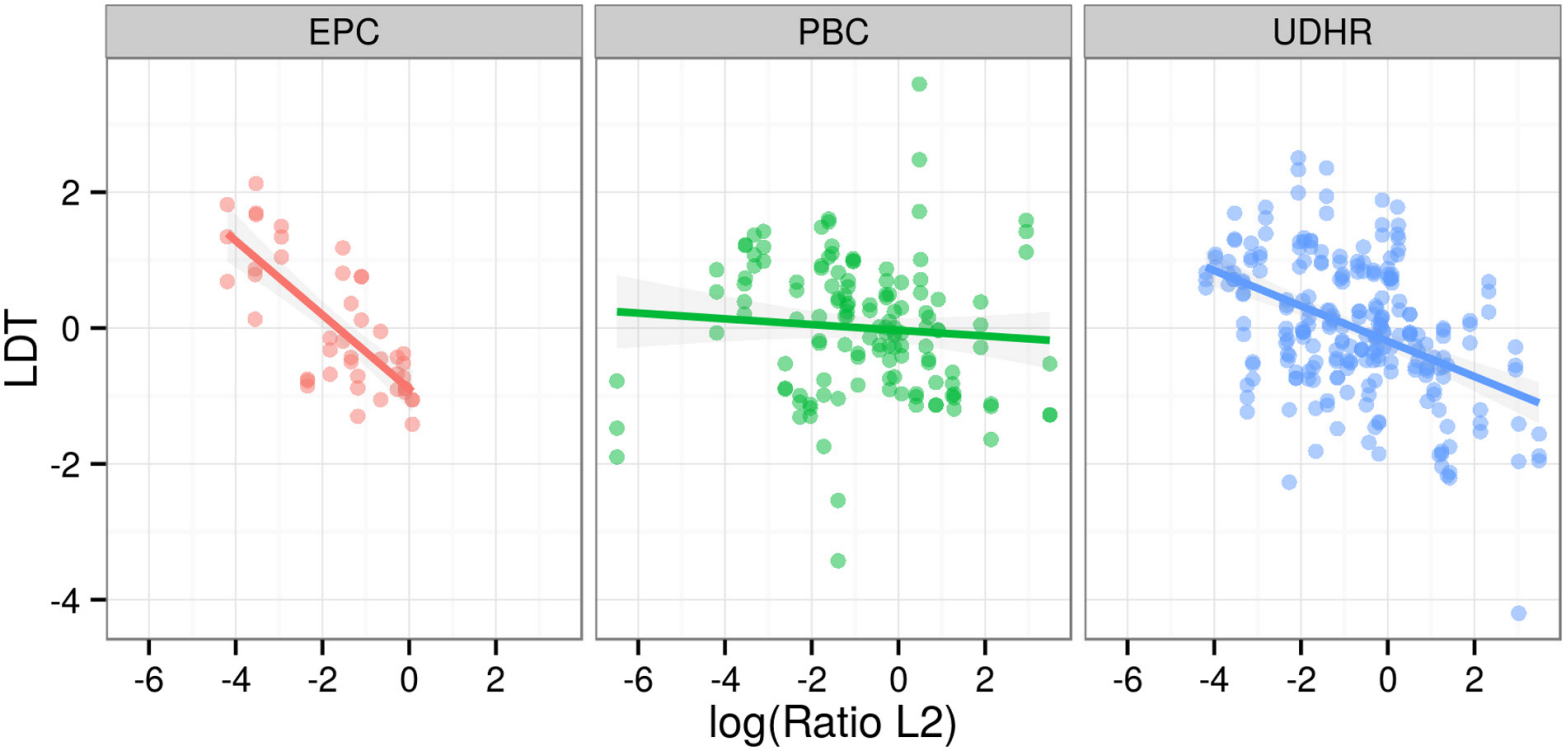
Regression plots by text types. Scatterplots of log-transformed ratios of L2 speakers versus LDTs facetted by text type. Lines represent linear models by families with 95% confidence intervals.

A visual way of establishing the significant result is to plot the relationship between log(RatioL2) and LDT for different families (Fig 7), different regions (Fig 8), different LDT measures (Fig 9) and different text types (Fig 10). These plots illustrate that the negative relationship holds for most families and regions, and for all three LDT measures as well as text types.

### Phylogenetic signal analyses

In Fig 4 we had Altaic and Indo-European languages as examples of clustering according to family membership. If this clustering holds for other families as well, we expect lexical diversities to generally have a strong phylogenetic signal. This is corroborated by the results for the *λ* analyses (Table 5).

**Table 5:** Results for the phylogenetic signal analysis (mean *λ*).

**Family**	**Text**	***α***	**H_w_**	**TTR**
Austronesian	UDHR	0.98	1	1
Austronesian	PBC	0.94	0.82	1
Bantu	UDHR	0.46	0.85	0.58
Indo-European	UDHR	1	0.64	1

For all three families for which enough phylogenetic tree information is available (Austronesian, Bantu, Indo-European) the LDT measures display *λ*-values above 0.5 and hence closer to 1 than to 0 (with the only exception being *α* for Bantu languages). There is a “deep” phylogenetic signal across the board. This is evidence that LDTs develop in parallel to the phylogenetic pathways reconstructed with cognate trees.

### Phylogenetic generalized least squares regressions

The PGLS regressions for 26 Indo-European languages again report relatively high *λ*-values, implying that a big part of the co-variance of lexical diversities can be explained by the phylogenetic relationships of the languages. However, despite the high *λ*-values, the ratio of non-native to native speakers is still a significant predictor for all three LDT measures (Table 6) after applying the Holm-Bonferroni correction.

**Table 6:** Results for PGLS.

**Dep. var.**	**Indep. var.**	**R^2^**	**coefficient**	**Standard error**	***λ***	**p-value**
ZM’s *α*	log(L2/L1)	0.15	0.07	0.03	0.67	0.03*
*H*_*w*_	log(L2/L1)	0.26	-0.28	0.1	0.57	0.003*
TTR	log(L2/L1)	0.17	-0.05	0.02	0.72	0.021*

* still significant after Holm-Bonferroni correction

## Discussion

The results of simple linear regression, mixed-effects regression and phylogenetic regression reveal that languages with higher non-native to native speaker ratios are (by trend) those languages with lower lexical diversities.

The simple linear regression yields a negative coefficient (-0.19) between L2/L1 ratio and scaled LDT, i.e. a plus of non-native speakers is associated with with a reduction of lexical diversity. The variation explained in the simple linear model amounts to 11% across 91 languages of 26 families. For the same sample of languages, the coefficient of a linear mixed-effects regression with families, regions, LDT measures, text types and ISO codes as random effects is significant (-0.28), indicating that the negative association between non-native speaker proportions and lexical diversities is not limited to a specific family, region, LDT measure or text type. In parallel to the mixed-effects regression, the PGLS regression for 26 Indo-European languages reveals that L2 ratio is still a significant predictor even after controlling for phylogenetic relatedness within that family.

Note, however, that there can still be families, regions, LDT measures or text types for which this relationship does *not* hold. For example, in Fig 7 languages are grouped by families. While for 5 of these overall 9 groups (Austronesian, Indo-European, Turkic, Creole and Other) the negative association holds, 2 display the inverse correlation (Atlantic, Uralic), and 2 do not display much of a relationship at all (Benue-Congo, Semitic). Likewise, 6 out of 9 regions (Fig 8) display the negative association (Europe, Greater Mesopotamia, Indic, Inner Asia, Oceania and Other), whereas for Southeast Asia the association seems inverted, and for South Africa and the African Savannah there is not much of a pattern at all. Hence, a conservative interpretation of the mixed-effects regression is that the negative association holds across *most* families and regions and across all LDT measures and text types (in our sample).

It is also important to point out that having L2/L1 ratios as *fixed effect* and adding families, regions, LDT measure, text types and ISO codes as *random effects* in a mixed-effects model means that they are considered to be different *kinds* of predictors. L2 ratios are seen here as a learning effect that (argueably) impacts the encoding strategy of a linguistic community *causally*. Families, regions, LDT measure, text type and ISO code, on the other hand, are just descriptive categories, i.e. means of binning or grouping. As such they are not supposed to causally explain lower or higher lexical diversities, they just categorize them and are taken into account as confounding factors.

Overall, the outcomes of all three models converge to show that L2 ratios can predict lexical diversities a) cross-linguistically, e.g. across different families, and b) within them same family. Conclusion a) is backed by the linear mixed-effects regression across different families, regions, text types and LDT measures. Conclusion b) is based on Indo-European languages only. In the following, we discuss the merits and limitations of our approach in more detail.

### What are we actually measuring as lexical diversity?

Given our definition of a word type, differences in lexical diversity can stem from a) inflectional marking (e.g. *sing, sang, sung*), b) derivation (e.g. *sing-er*), c) prefixes (e.g. *re-consider*), d) compounding (e.g. *snowwhite*), e) differences in the base vocabulary (loanwords, neologisms) and f) variation in orthography (e.g. *neighbor* and *neighbour*). This begs the question which factor is most important, and hence, what difference we are actually measuring using LDT.

A recent study [56] has shown that in the history of English LDT has systematically decreased. Namely, the LDT for the Old English (OE) version of the Book of Genesis was 23% higher than the LDT of the Modern English (MnE) parallel translation, whereas the deviation between different texts of the same period (either OE or MnE) was only 1–2%. Further analyses suggested that the bigger difference in LDTs between OE and MnE derive from the loss of inflectional marking.

These observations align with earlier studies arguing that the parameter *α* of Zipf’s law decreases in children’s speech when they learn to use a wider range of vocabulary and apply inflections more productively [57], that parameter *α* is lower for languages with more grammatical marking [58–60], that it is lower for texts un-lemmatized compared to lemmatized texts [24], and that LDT can be increased by merging words in a simplified grammaticalization model [61].

Overall, though all the factors a)-f) are involved in the variation of LDT values, based on earlier studies it is reasonable to assume that especially the factors under a)-d) play a predominant role for variation in numbers of word forms.

### Non-native speakers and lexical diversity

According to theories relating to language contact [10–12, 14–16, 62] non-native speakers in a population can bias the shared language towards exhibiting less morphological elaboration. Several historic and sociolinguistic studies have pioneered this hypothesis on qualitative grounds [10, 11, 14, 62]. For example, it is argued that the degree of inflection loss and levelling is considerably lower for low-contact Germanic languages such as Faroese and Icelandic than for high-contact varieties such as English and Dutch [14, p. 72]. Especially in the history of English the assimiliation of non-native speakers of Scandinavian populations [11, p. 91], Late British speakers [14, p. 55], and French-speaking Normans were named as potentially driving a reduction in morphological elaboration (see [16] for a more detailed discussion with reference to case marking).

These qualitative studies of the histories and properties of specific languages are backed by quantitative studies that use statistical models to link population size [12] and non-native speaker ratios [15, 16] with less morphological marking across many languages. Given that less morphological marking is tightly linked with lower lexical diversities, the qualitative and quantitative explanations elaborated by the aformentioned studies also constitute the most promissing explanation for the results reported in the current study. This is not to say, of course, that there cannot be any other “lurking variables” and potential alternative explanations for variance found in LDTs.

### Synchronic data and diachronic implications

The study presented here is mainly synchronic, i.e. the associations between a) recent properties of parallel texts and b) recent numbers of non-native speakers are a cross-section of diachronic processes. It is reasonable to ask whether conclusions about diachronic processes can be reached based on such an analysis.

However, an independent study on OE and MnE parallel translations of the Book of Genesis demonstrated that reduced lexical diversity can be the outcome of changes in a language over historical time, and that these changes can be quantified using frequency distributions [56]. In addition, there is evidence that the mean population ratios between languages of the same areas (Africa, Eurasia, Australia and New Guinea, and the Americas) can be extrapolated into the past [63] by several thousand years (with diminishing acuracy). Of course, for certain languages, non-native speaker ratios fluctuate over time due to migration and trade routes. However, across 91 languages we expect fluctuations to average out. Moreover, the phylogenetic methods used allow us to infer pathways of evolution and how they are related to the relevant predictor variables (L2 ratio) in a family tree. Hence, we observe synchronic results of diachronic processes that have potentially affected the languages under investigation in the past.

### Parallel texts as doculects

The EPC, UDHR and PBC are highly specified texts of a certain genre, register and style, i.e. so-called doculects. Such doculects represent languages in a rather indirect fashion [64, 65]. An optimal solution would be to compile balanced corpora of parallel texts for hundreds of languages, but such a balanced corpus is currently not available.

Having said that, there is evidence that systematic variation in lexical diversity is not confined to our parallel texts, but reflected in frequency distributions of various parallel and non-parallel texts [56, 58–60, 64, 66].

Moreover, as Fig 10 illustrates, the correlation between LDT and L2 ratios holds across all three parallel text corpora, independent of text size (UDHR, ca. 2000 words per language, PBC ca. 20000 words per language, EPC ca. 7mio words per language) as well as genre (legal text, religious texts, written speeches). This suggests that the effect is robust and extrapolates beyond the doculects used here.

### Which languages have the best information encoding strategy?

Neither previous studies on language “simplification” [12–16] nor the present work makes any claims as to whether lexically rich or poor languages are more efficient or less efficient overall, or “better” or “worse” communicative systems in an absolute sense. It has been argued elsewhere [67–70] that the assignment of complex meanings to constructions (e.g. fixed word orders) can compensate for a lack of lexical diversity (e.g. less inflectional variants). These claims are independent of the findings reported in this study, that languages that recruited significant numbers of adult non-native speakers in their histories are more likely to exhibit low lexical diversity. However, the results do indicate that languages as communication systems adapt to the learning constraints of speaker populations.

### Are all languages directly comparable?

Our analyses include Creole languages. Because of their abrupt creation by L2 speakers, it might be argued that Creole languages are not a coherent group comparable to a language family like Indo-European. However, it is equally plausible that the same L2 learning pressures that most strongly shape Creole languages are at play in historical language change of other languages as well, albeit to a lesser extent (see also [11, 14]). From this perspective, the difference between Creole languages and other language groupings is a matter of degree, rather than categorical. Including them as a sub-group instead of excluding them categorically can therefore only help to better understand the pressures that shape languages over time.

### Correlation is not causation

Spurious correlations are a recurring problem in studies of sociolinguistic variation [71, 72], where independent evidence can help to support claims of a causal relationship. In the present case, a causal link between non-native learning and reduction of lexical diversity is supported by two areas of research:

1. Qualitative sociolinguistic studies are replete with examples of non-native speakers reducing morphological marking and hence lexical diversity over time [11, 13, 14, 62] and these are backed by quantitative evidence [12, 15, 16].
2. In the context of measuring lexical diversity for teaching purposes it has been shown that L2 learners of French [18] and English with various L1 backgrounds [17] produce output of lower lexical diversity compared to native speakers.

We therefore emphasise the converging evidence from qualitative and quantitative, diachronic and synchronic studies showing that the presence of significant numbers of non-native speakers systematically lowers the likelihood of preserving lexically rich encoding systems.

## Conclusion

Languages with more non-native speakers tend to have lower lexical diversities, i.e. fewer word forms and higher word form frequencies. This trend holds across different language families, regions, measures, and text types. In other words, non-native language learning and usage emerges as important factor driving language change and evolution besides native language transmission.

Since non-native language learners are prone to reduce manifold word forms to a smaller set of base forms, it is natural that they shape the lexical encoding strategies of the next generation of learners. It is not clear, and not particularly relevant for our approach, whether the resulting lower lexical diversity results in a “better” or “worse” encoding strategy. The picture that emerges, however, suggests that in the long run, languages as encoding systems can adapt to sociolinguistic pressures, including those determined by learning abilities and constraints of their speakers. This finding can help to disentangle the complex relationship between language learning, language typology and language change. As a result, theories of language evolution should take into account the co-evolution of population structure, human learning abilities and language structure.

## Supporting Information

S1 File Word type definition and writing systems. (PDF)

S2 File Maximum likelihood (ML) estimation. (PDF)

S3 File ZM-parameters, entropy and type-token ratios as measures of lexical diversity. (PDF)

S4 File Checking assumptions of statistical models. (PDF)

S5 File Phylogenetic regressions with BayesTraits. (PDF)

S1 Table List of ML estimated parameters, entropies and type-token ratios for 647 languages of the EPC, PBC and UDHR. iso_639_3: ISO 639-3 codes as language identifier; fileName: name of the file in the original parallel text corpus; language: language name; LDT: unscaled lexical diversity; LDT: same as LDT but with ZM’s *α* inverted by (1- *α*); LDTscaled: scaled LDTinv; measure: LDT measure used (a, H, TTR); text: parallel text corpus (EPC, PBC, UDHR); genus_wals: language genus taken from WALS; family_wals: language family taken from WALS; latitude: latitude taken from WALS; longitude: longitude taken from WALS;
(CSV)

S2 Table Ratios of non-native speakers per language. Subsample of 91 languages for which both L2 information and lexical diversity estimates are available. This subsample was used for statistical modeling. iso_639_3: ISO 639-3 codes as language identifier; fileName: name of the file in the original parallel text corpus; Language: language name; Stock: language family taken from AUTOTYP database; Stock_coarse: language families with more than 5 members in the sample; Region: region taken from AUTOTYP database; Region_coarse: language regions with more than 5 members in the sample; L1_speakers: estimated numbers of native speakers; L2_speakers: estimated numbers of non-native speakers; RatioL2: ratio of L2 to L1 speakers; PercL2: percentage of L2 speakers; LDT: unscaled lexical diversity; LDT: same as LDT but with ZM’s *α* inverted by (1- *α*); LDTscaled: scaled LDTinv; measure: LDT measure used (a, H, TTR); text: parallel text corpus (EPC, PBC, UDHR); genus_wals: language genus taken from WALS; family_wals: language family taken from WALS;
(CSV)
