# Supplementary material for: Adaptive Communication: Languages with More Non-Native Speakers Tend to Have Fewer Word Forms
Source: PLoS One. 2015 Jun 17;10(6):e0128254. doi: 10.1371/journal.pone.0128254 (PMC4470635; doi:10.1371/journal.pone.0128254)
Supplement: S1 File — (PDF) [file pone.0128254.s001.pdf]

S1 File

Word type definition and writing systems

Differences in writing systems are of some importance to us for at least two reasons:

1) Not all writing systems separate words by white spaces (e.g. logographic writing systems as in many Sino-Tibetan languages). To our knowledge this rules out 23 of the languages for which translations of the UDHR are currently available and another 63 of the PBC. In order to filter out the untokenizable languages we checked frequency lists for outstanding patterns (e.g. frequencies < 30 for even the highest frequency items). In borderline cases we consulted Daniels & Bright (1996) to decide whether our approach is feasible for a specific language. For some of the most widespread writing systems, i.e. Latin, Cyrillic, Devanagari and Arabic we had native speakers check word lists for misclassified items. Overall, very few instances of misclassified items were found. One example was a punctuation mark in a Devanagari script that was not filtered out and misinterpreted as a single letter word. Note, however, that such misclassification of single items is very unlikely to change frequency distributions enough to render significantly different results for the Zipf-Mandelbrot parameters, Shannon entropy or type-token ratios.

2) For some languages translations are available in two different scripts. For example, Azerbaijani, Turkmen, Serbian, Bosnian and Uzbek are available in both Latin and Cyrillic scripts. Comparing their lexical diversity values is informative as to whether we expect writing systems to have an impact on our lexical diversity measures. Figure S1 suggests that they do (albeit to a negligible extent). It plots ZM parameters, entropies and type-token ratios for the Latin versions versus the Cyrillic versions of five languages. If both versions always had the same lexical diversity values, they would fall on the straight line. However, for all languages the Latin versions have slightly higher α, and for all languages except Turkmen the Latin languages have slightly lower entropies and TTRs. This suggests, for example, that Latin script gives rise to slightly lower lexical diversities than Cyrillic script.

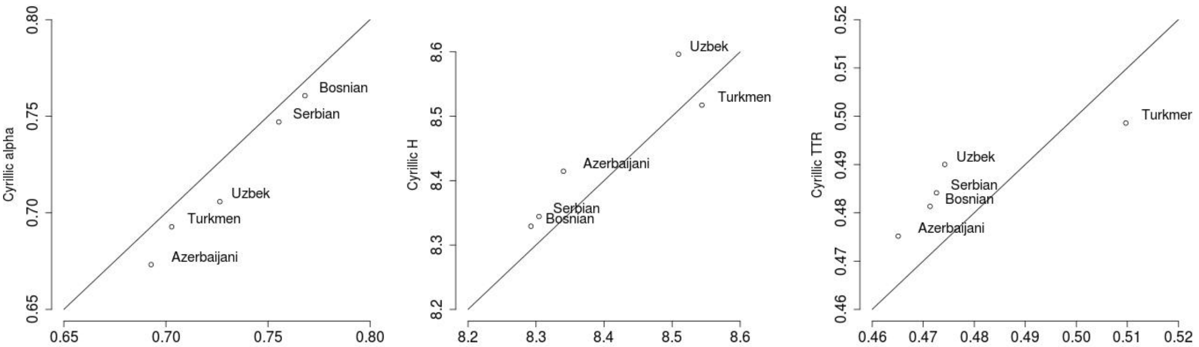

S2 Figure. Writing systems. ZM parameter α (left panel), entropies (*H*) (middle panel) and TTRs (right panel) for the same languages in different scripts (i.e. Latin and Cyrillic). The line marks equivalent values.

Note, however, that even in the most extreme case (Uzbek TTRs) this difference only amounts to 0.015 which is ca. 3%.

Daniels, P. T., & Bright, W. (Eds.). (1996). *The world's writing systems*. New York/ Oxford: Oxford University Press.
