# Supplementary material for: Adaptive Communication: Languages with More Non-Native Speakers Tend to Have Fewer Word Forms
Source: PLoS One. 2015 Jun 17;10(6):e0128254. doi: 10.1371/journal.pone.0128254 (PMC4470635; doi:10.1371/journal.pone.0128254)
Supplement: S2 File — (PDF) [file pone.0128254.s002.pdf]

S2 File

Maximum likelihood (ML) estimation

We used the *likelihood* package (Murphy, 2013) in *R* (R Core Team, 2013) implementing a simulated annealing procedure to estimate the parameters for best fitting curves. The pre-defined scientific model is the discrete and finite version of the ZM distribution in Equation (i):

$$P(X = r) = \frac{C}{(\beta + r)^{\alpha}} \quad C > 0,\ \alpha > 0,\ \beta > -1,\ r = 1, \ldots, k. \quad \text{(i)}$$

In this equation *r* is the rank of a word type in a frequency distribution, *k* is the number of different word types, α and β are parameters and *C* is a normalizing constant defined as:

$$C = \left(\sum_{r=1}^{k} (\beta + r)^{-\alpha} \right)^{-1}.$$

Parameters α and β are to be estimated.

Following Izsák (2006) we assume that the frequencies *f* of words in a random sample $X = (f_1, f_2, \ldots, f_k)$ of size *n* will be multinomially distributed, which renders the likelihood function in Equation (ii):

$$L(\theta \mid X) = \frac{n!}{f_1!\, f_2! \ldots f_k!} \prod_{i=1}^{k} p_{\theta,i}^{f_i}. \quad \text{(ii)}$$

Plugging Equation (i) into Equation (ii) then renders the likelihood function to be maximized:

$$L(\theta \mid X) = P(f_1, f_2, \ldots, f_k, \alpha, \beta) = \frac{n!}{f_1!\, f_2! \ldots f_k!} \prod_{i=1}^{k} \left(\frac{C}{(\beta + r)^{\alpha}} \right)^{f_i}. \quad \text{(iii)}$$

To find the optimal parameters for (iii) the *likelihood* package uses an annealing algorithm (see Murphy, 2013) that systematically searches the parameter space rather than numerically solving the maximization problem. We restricted the cycles of the annealing function to a maximum of 10000. Also, we followed Izsák (2006: 112-113) in giving initial parameters approximated by means of a linear regression model.

References

Izsák, J. (2006). Some practical aspects of fitting and testing the Zipf-Mandelbrot model: A short essay. *Scientometrics*, *67*(1), 107–120. doi:10.1556/Scient.67.2006.1.7

Murphy, L. (2013). R package “likelihood”: Metods for maximum likelihood estimation. Retrieved from cran.r-project.org/web/packages/likelihood/index.html

R Core Team. (2013). R: A language and environment for statistical computing. Vienna, Austria: R Foundation for Statistical Computing. doi:ISBN 3-900051-07-0
