# Supplementary material for: Adaptive Communication: Languages with More Non-Native Speakers Tend to Have Fewer Word Forms
Source: PLoS One. 2015 Jun 17;10(6):e0128254. doi: 10.1371/journal.pone.0128254 (PMC4470635; doi:10.1371/journal.pone.0128254)
Supplement: S3 File — (PDF) [file pone.0128254.s003.pdf]

S3 File

ZM-parameters, entropy and type-token ratios as measures of lexical diversity

We compared the “responsiveness” of our lexical diversity measures to changes in frequency distributions by using a) parallel texts (Book of Genesis) in Old English and Modern English and b) the same text in Modern English and its lemmatized counterpart (i.e. stripped of all morphological marking). Note, that the OE text has richer morphological marking and hence a higher lexical diversity which was reduced towards Modern English (Bentz, Kiela, Hill, & Buttery, 2014). The lemmatized version of the Modern English text is then even further reduced in terms of lexical diversity due to the reduction of morphologically marked forms to lemmas. Hence, comparing values of our lexical diversity measures for these parallel texts can help us determine how sensitive they are to controlled changes in word frequency distributions. The results are summarized in Table S2.

As illustrated by the rates of change for parallel texts in Table S2, TTR is most sensitive to changes in word frequency distributions and hence lexical diversity, followed by ZM’s α and Shannon entropy H, which is the least sensitive (e.g. percentage-wise it changes more than five times less compared to the type-token ratio, namely 8% versus 44%).

Table A. Comparing lexical diversity measures for parallel texts

Texts	LDT measure		
	α	H	*TTR*
Old English	1.03	9.09	0.16
Modern English	1.22	8.39	0.09
**Rate of change**	**16%**	**8%**	**44%**
Modern English	1.22	8.39	0.09
ME (lemmatized)	1.29	8.11	0.07
**Rate of change**	**5%**	**3%**	**22%**

References

Bentz, C., Kiela, D., Hill, F., & Buttery, P. (2014). Zipf’s law and the grammar of languages: A quantitative study of Old and Modern English parallel texts. *Corpus Linguistics and Linguistic Theory*.
