# Supplementary material for: Adaptive Communication: Languages with More Non-Native Speakers Tend to Have Fewer Word Forms
Source: PLoS One. 2015 Jun 17;10(6):e0128254. doi: 10.1371/journal.pone.0128254 (PMC4470635; doi:10.1371/journal.pone.0128254)
Supplement: S4 File — (PDF) [file pone.0128254.s004.pdf]

# S4 File

## Checking assumptions of statistical models

### Simple linear model

We used the function *lm()* in R (R Core Team, 2013) for building simple linear models of L2 ratios predicting lexical diversity measures. Linear models require the following assumptions to be met:

*1) Linearity*: We assume that a linear relationship holds between our measure of lexical diversity (LDT scaled) and our measure of language contact (L2 ratio). That this assumption is generally met can be seen in Figure A. The only significant divergence from linearity is caused by three points of the language Nuer (nus) of South Sudan. Since all three outliers stem from the same language, this seems to be an idiosyncrasy rather than a general non-linear trend.

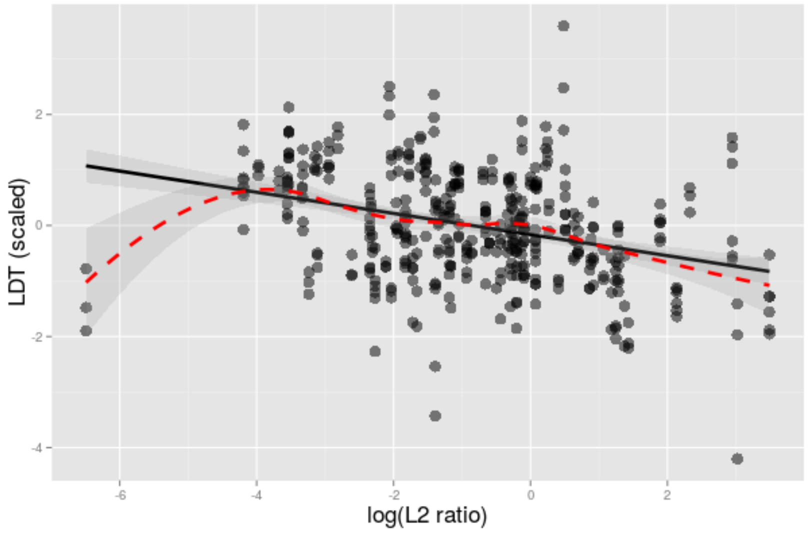


**Figure A. Linearity assumption.** Linear relationship between LDT and ratio of L2 speakers. The linear model for all lexical diversity measures (black lines) and their 95% confidence intervals (transparent grey) generally overlap with a local smoother of the type "Loess" (red dashed line) which is highly sensitive to non-linearities in the data.

2) *Normality*: Another model assumption is that the errors (residuals) are approximately normally distributed. This assumption can be checked with reference to Figure B.

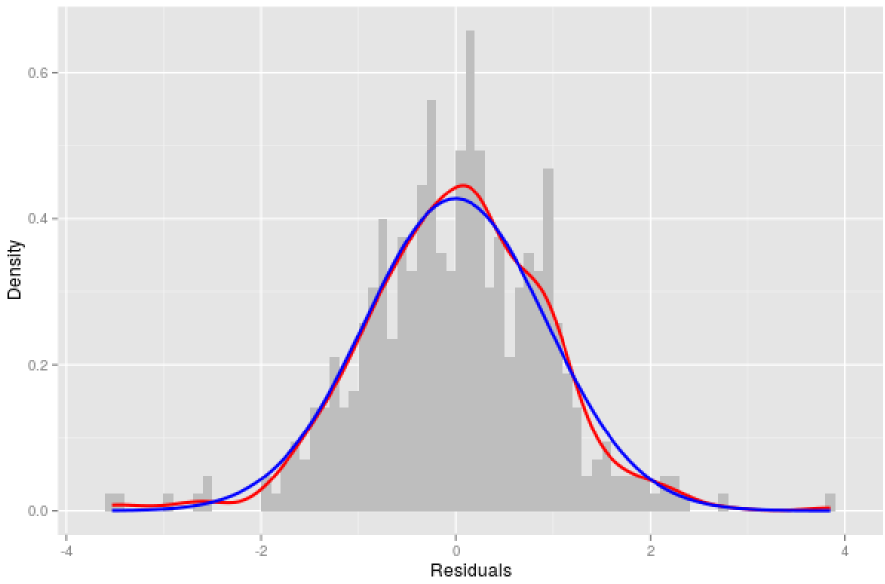


**Figure B. Normality assumption.** Distribution of residuals for the simple linear model. The approximated empirical density curves (red) follow closely the theoretical density curves (blue).

3) *Homoscedasticity*: It is assumed that the variation of residuals is relatively uniform across fitted values, i.e. the deviation from fitted values should not exhibit any clear patterns. To check this, we plot fitted values of our linear models versus their residuals (Figure C). The confidence intervals of a straight line through these data points should always include the zero line.

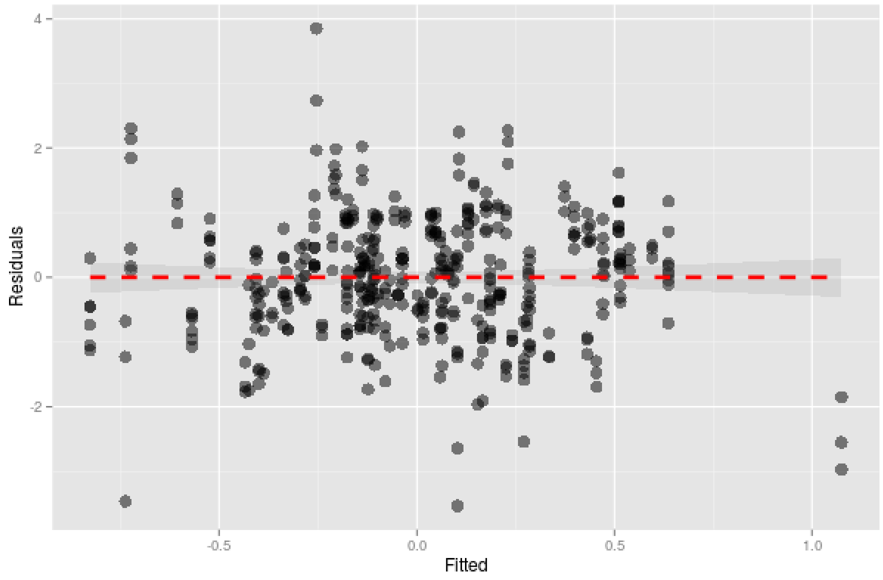


**Figure C. Homoscedasticity assumption.** Plot of fitted values versus residuals for  the simple linear model. The red dashed lines indicate a linear model with 95% confidence intervals (transparent grey).

**Mixed-effects model**

We used the package *lme4* (Bates, Maechler, & Bolker, 2012) in R (R Core Team, 2013) for linear mixed-effects modeling. The mixed-effects model requires the same assumptions as the simple linear model to be met or at least approximated (Baayen, 2008; Jaeger, Graff, Croft, & Pontillo, 2011; Winter, 2013).

1) *Linearity*: We assume that a linear relationship holds between our measures of lexical diversity and our measure of language contact (L2 ratio). This assumption is the same as for the simple linear model. That it is met was shown in Figure A.

2) *Normality*: In Equation (11) we made the assumption that $\varepsilon_{frmti} \sim N(0,\sigma^2)$, i.e that the errors or residuals are distributed normally around their mean. As can be seen in Figure D this assumption is met, though residuals cluster somewhat closer to the mean than expected in a perfectly normal distribution.

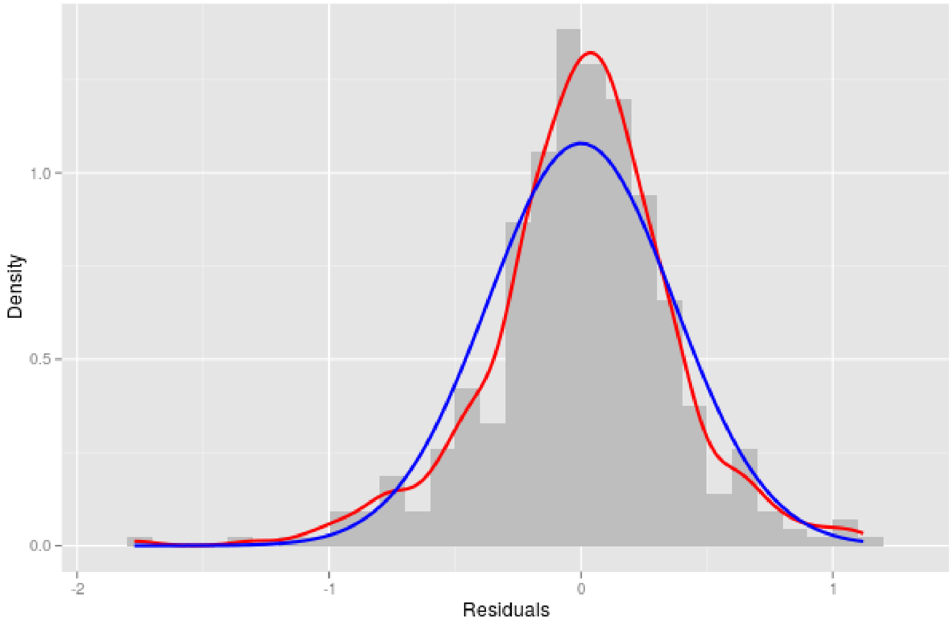


**Figure D. Normality assumption for mixed-effects model.** Distribution of residuals. The approximated empirical density curve (red) follows roughly the theoretical density curve (blue).

3) *Homoscedasticity*: It is assumed that the variation of residuals is relatively uniform across fitted values, i.e. the deviation from fitted values should not exhibit any clear patterns. To check this we plot fitted values of our model versus residuals (Figure E).

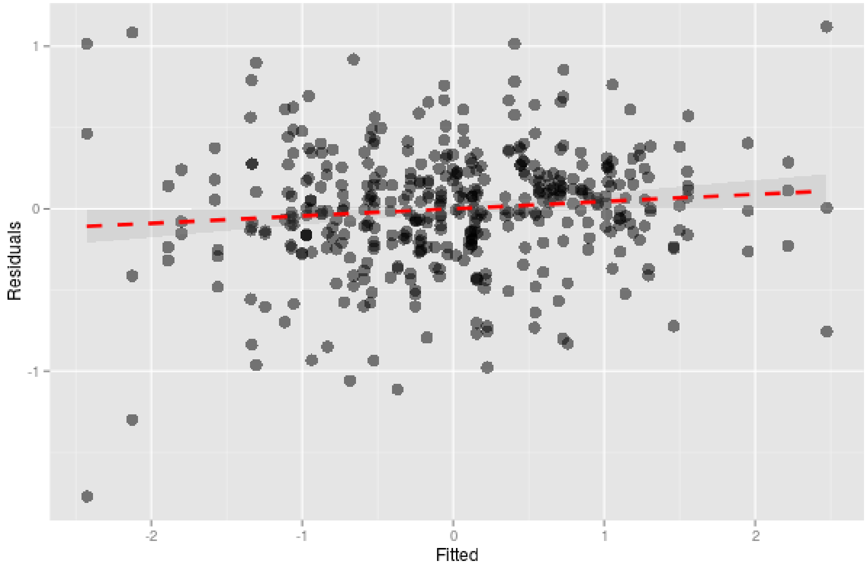


**Figure E. Homoscedasticity assumption for mixed-effects model**. Plot of fitted values versus residuals. The red dashed lines indicate a linear model with 95% confidence intervals (transparent grey).

Note, that the 95% confidence interval just falls above and below the zero line, which suggests heteroscedasticity, albeit very minor. Note, that Jaeger et al. (2011: 293) would also recommend checking residual plots for subgroups of families and regions. However, due to data sparsity this will not be informative in our case.

## References


Baayen, H. R. (2008). Analyzing linguistic data: A practical introduction using R. Cambridge: Cambridge University Press. Retrieved from http://cran.r-project.org/package=languageR.

Bates, D., Maechler, M., & Bolker, B. (2012). lme4: Linear mixed-effects models using S4 classes. Retrieved from http://cran.r-project.org/package=lme4

Jaeger, T. F., Graff, P., Croft, W., & Pontillo, D. (2011). Mixed effect models for genetic and areal dependencies in linguistic typology. *Linguistic Typology*, *15*, 281–320. doi:10.1515/LITY.2011.021

R Core Team. (2013). R: A language and environment for statistical computing. Vienna, Austria: R Foundation for Statistical Computing. doi:ISBN 3-900051-07-0

Winter, B. (2013). Linear models and linear mixed effects models in R with linguistic applications. Retrieved from http://arxiv.org//pdf/1308.5499.pdf
