# Supplementary material for: Adaptive Communication: Languages with More Non-Native Speakers Tend to Have Fewer Word Forms
Source: PLoS One. 2015 Jun 17;10(6):e0128254. doi: 10.1371/journal.pone.0128254 (PMC4470635; doi:10.1371/journal.pone.0128254)
Supplement: S5 File — (PDF) [file pone.0128254.s005.pdf]

S5 File

Phylogenetic regressions with BayesTraits

To illustrate the association between lexical diversity and the amount of non-native (L2) speakers at the level of genera while controlling for phylogenetic dependencies, Phylogenetic Generalized Least Squares (PGLS) regression analyses (Pagel, 1997: 340ff.) were carried out for a sub-sample of 26 Indo-European languages. To conduct the PGLS regression we used the phylogenetic comparative method *Continuous* implemented in the BayesTraits package from http://www.evolution.rdg.ac.uk/BayesTraits (Pagel, 1997, 1999). BayesTraits can perform both maximum-likelihood (ML) and Bayesian analyses.

We used a Bayesian approach in order to estimate the posterior probability distributions of the parameters of interest to the comparative question (the association between lexical diversity and the amount of non-native (L2) speakers). Bayesian approaches are preferred in evolutionary biology because they allow for the setting of data-driven priors and the estimation of a posterior probability of parameters.

This enables the researcher to specify the evolutionary model required as well as finding distributions of the regression solution rather than a single solution. The posterior probability of a parameter (such as the regression coefficients) is a number proportional to its likelihood of having produced the observed data. It represents the probability of the parameter value given the dataset and the model of trait evolution (Ronquist, 2004). Posterior probability distributions provide information about the degree of statistical uncertainty in the estimation of the parameters of interest. Combined with the usage of a sample of phylogenetic trees rather than a single phylogenetic tree, the analyses find regression solutions that are not dependent on a single hypothesis regarding the exact genealogical relations of the languages. In addition, different models of trait evolution can be tested and compared, yielding results that are not dependent on any specific or underspecified model of trait evolution.

Given the phylogenetic tree sample and the comparative data, *Continuous* uses a Brownian motion model of evolution implemented within a generalised least squares (GLS) approach (Pagel, 1997, 1999). Under this model, the continuous trait is modelled to change with a mean change of zero and a fixed variance in each infinitely small unit of time. In each of these ‘units of time’, change is assumed to take place independently, not taking into account changes on previous or upcoming parts of the tree. The variance of change is in direct proportion to the amount of time the languages have been evolving. Languages that have diverged furthest from the root have the largest variance and are thus the least reliable to use for inferences regarding the behaviour of the root. Closely related languages tend to behave similarly as they share most of their genealogical history as indicated by the phylogenetic tree. Within the GLS approach, the Brownian motion model of evolution can be implemented as a regression analysis as follows:

$$y = \alpha + \beta x + \varepsilon,$$

in which a feature y is dependent on a feature x, α is the intercept of the regression line, β is the slope, and ε is the error term. The GLS implementation allows to estimate the regression coefficient β while taking into account the genealogical relationships of the languages as

indicated by the phylogeny. Phylogenetically determined non-independence among the languages is accounted for by a matrix of the expected co-variance amongst languages, which is derived from the phylogenetic tree. It is also possible to transform and scale the phylogeny to test the adequacy of the underlying model of evolution and to find the best fit of the phylogeny to the data. In these analyses, we estimated the parameter λ (lambda) to test for phylogenetic signal, and thus to test whether this analysis was adequate.

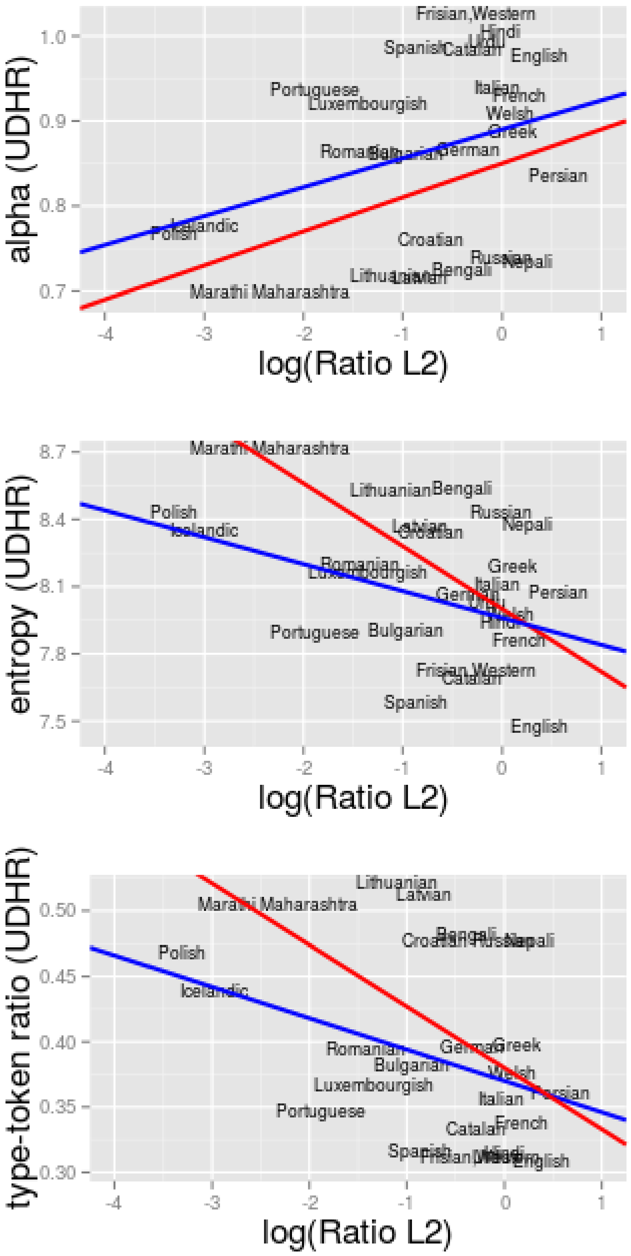

Figure A. Phylogenetic regression plots. Simple linear regression models (blue) and PGLS regression models (red) for the relationship between ratio of L2 speakers (logarithmically transformed) and ZM's α (left panel), entropy (middle panel) and type-token ratios (right panel).

The Bayesian MCMC analysis as implemented in *Continuous* estimates the posterior

probability distribution of the relevant parameters, the regression coefficients and their error terms (Pagel & Meade, n.d.). It is impossible to compute posterior probabilities comprehensively due to the amount of computing time that would take, and therefore they are usually inferred by a MCMC (Markov chain Monte Carlo) sampling algorithm. The MCMC chain explores the parameter space widely, making random changes to the parameters and assessing their effect on the likelihood while using the sample of trees during a large number of consecutive iterations. Whether the chain jumps to a new state is determined by the likelihood of the proposed state: states with a higher likelihood than the current state are always accepted, while states with a lower likelihood are accepted in accordance with how much lower their likelihood is. After an initial amount of iterations of 'burn-in', the chain becomes stationary in terms of likelihood, and parameter values are sampled into the posterior distribution. In this way, the posterior distribution contains a sample of parameters in which those most highly supported have been sampled the most.

For each of the different measures of lexical diversity, i.e. Shannon entropy H, Zipf-Mandelbrot parameter α and type-token ratio (TTR), one MCMC chain was run for 2×10^9 iterations. The regression coefficients were sampled from the chain into the posterior distribution every 1,000,000th iteration. A burn-in of 5×10^8 was taken from the beginning of the chain. This leaves a posterior distribution of 1,500 estimations of the regression solution. The medians of this posterior probability distribution of regression coefficients are given in the main text. The coefficients were tested for statistical significance by taking the ratio of the time each coefficient is smaller than 0 divided by the time each coefficient is bigger than 0 (Pagel and Meade, n.d.).

A graphical comparison between coefficients for the PGLS regressions and simple linear regressions can be seen in Figure S7.

References

Pagel, M. (1997). Inferring evolutionary processes from phylogenies. *Zoologica Scripta, 26*(4), 331–348.

Pagel, M. (1999). Inferring the historical patterns of biological evolution. *Nature, 401*, 877–884.

Pagel, M., & Meade, A. (n.d.). Bayes Traits V2. Retrieved from www.evolution.rdg.ac.uk/Files/BayesTraitsV2Manual(Beta).pdf

Ronquist, F. (2004). Bayesian inference of character evolution. *TRENDS in Ecology and Evolution, 19*(9), 475–481.
